# Hybrid prosthesis in frozen elephant trunk procedures for hereditary thoracic aortic diseases: a 14-year single-aortic center experience

**DOI:** 10.1186/s13019-025-03738-7

**Published:** 2025-11-27

**Authors:** Jens Brickwedel, Lennart. Bax, Till Joscha Demal, Tilo Kölbel, Yskert von Kodolitsch, Bernadette E. Bessick, Hermann Reichenspurner, Christian Detter

**Affiliations:** 1 Department of Cardiovascular Surgery, University Heart and Vascular Center Hamburg, Martinistraße 52, 20246 Hamburg, Germany; 2 Department of Vascular Medicine, University Heart and Vascular Center Hamburg, Hamburg, Germany; 3 Department of Anatomy, Medical School Hamburg, University of Applied Sciences and Medical University, Am Kaiserkai 1, 20457 Hamburg, Germany; 4 Clinic for Thoracic and Cardiovascular Surgery, Section of Vascular surgery, Heart and Diabetes Centre North Rhine Westphalia, Bad Oeynhausen, Germany; 5 German Center for Cardiovascular Research (DZHK), Berlin, Germany

**Keywords:** Aortic arch, Frozen elephant trunk, Hybrid stent graft, Genotype, Hereditary thoracic aortic disease

## Abstract

**Background:**

There is a lack of data regarding the use of hybrid stent graft prostheses in patients with hereditary thoracic aortic disease (HTAD) involving the aortic arch and proximal descending aorta. This retrospective analysis aimed to evaluate the short- and mid-term outcomes of hybrid stent-graft prostheses in Frozen Elephant Trunk (FET) procedures for patients with HTAD, with a particular focus on its safety and feasibility.

**Methods:**

A total 280 patients who underwent FET procedures between October 2010 and November 2024 were retrospectively analysed in compliance with the 2024 EACTS/ STS recommendations for shared decision-making within the multidisciplinary aortic team. Among them, 51 patients had genetically confirmed HTAD (Marfan syndrome (FBN1), Loeys-Dietz syndrome (TGFBR1, TGFBR2, SMAD3, TGFB2), vascular Ehlers-Danlos syndrome (COLSA1), and non-syndromic HTAD (ACTA2, MYH11, MYLK)). The Thoraflex™ prosthesis was implanted in 50 of the 51 patients. Short- and mid-term outcomes were assessed descriptively. Survival and subsequent thoracic aortic intervention rates were analysed using the Kaplan-Meier method.

**Results:**

The overall 30-day mortality was 2.0% (*n* = 1). Perioperatively, permanent neurological deficit was 3.9% (*n* = 2), with minor disability on the modified Rankin Scale (mRS 1 and 2). There were no instances of paraplegia. The median follow-up was 4.0 years. The 1-, 3- and 5-year overall survival rate was 93.9%, 90.6%, and 90.6%, respectively. Freedom from subsequent aortic interventions was at 1, 3, 5 years 55.8%, 45.6%, and 33.1%. Early device-related complications occurred in 7 patients (13.7%), including intraluminal FET thrombosis in 4 patients (12.5%) and distal stent graft-induced new entry in 3 patients (9.4%). Mid-term device-related complications occurred 2 patients (4.3%).

**Conclusions:**

Hybrid stent graft prostheses can be safely implanted with the FET technique in elective and acute HTAD patients with arch and proximal descending aortic disease. Our single-center short- and mid-term outcomes are encouraging, but long-term durability and efficacy are not yet established. This warrants multi-center studies with extended follow-up.

**Supplementary Information:**

The online version contains supplementary material available at 10.1186/s13019-025-03738-7.

## Background

Hereditary thoracic aortic diseases (HTAD) represent around 20% of all thoracic aortic aneurysms and dissections. HTAD includes Marfan syndrome (MFS), Loeys-Dietz syndrome (LDS), vascular Ehlers-Danlos syndrome (vEDS), and non-syndromic HTAD (nsHTAD). Prognosis in HTAD varies, based on the underlying gene harboring the pathogenic variants, as outlined in current guidelines by the European Association for Cardio-Thoracic Surgery (2024 EACTS/STS) [1].

The Frozen Elephant Trunk (FET) procedure is one of the surgical options available for treating aortic arch pathologies. It enables one-stage repair of aortic arch pathologies and facilitates subsequent thoracic aortic interventions, also reported in patients with MFS [2, 3]. The EACTS guidelines provide a level C, class 1 or 2a recommendation for FET implantation in aortic arch pathologies, such as aneurysms and aortic dissections with anticipated distal complications. While there are well defined surgical recommendations for aortic root replacement, particularly for the different HTAD pathologies, guidelines for managing aortic arch pathologies in HTAD remain non-specific.

Currently, short- and mid-term data regarding the benefits and complications associated with using commercially available hybrid stent graft prostheses in the FET procedure for replacing the aortic arch and proximal descending thoracic aorta, are predominantly available for patients with MFS. The first report of hybrid prostheses using the FET technique in patients with MFS appeared in 2013.^4^ Data for all other HTAD pathologies remain very limited. There are indications that placing stent grafts within Dacron grafts in patients with HTAD is feasible [2, 5, 6]. This retrospective, single-center observational cohort study of consecutive patients evaluates the short- and mid-term outcomes of hybrid stent-graft implantation using the FET technique for aortic arch and proximal descending thoracic replacement in patients with HTAD, with the focus on its safety and feasibility.

## Method

### Patients and study design

This retrospective observational cohort study was conducted at the Hamburg Aortic Center and included all consecutive patients who underwent aortic arch and proximal descending aortic repair using the frozen elephant trunk (FET) technique between October 2010 and November 2024. A total 280 patients who underwent FET procedures in compliance with the 2024 EACTS/STS recommendations for shared decision-making within the multidisciplinary aortic team were retrospectively analysed. Among these, 51 patients were diagnosed with genetically confirmed HTAD through a comprehensive, multidisciplinary evaluation based on multifactorial criteria. To minimize underdiagnosis, genetic testing decisions are guided by the GHENT criteria. Indications include age < 60 years, syndromic features (MFS, LDS, vEDS) or specific signs (e.g., dural ectasia, aortic root dilation), and a personal or family history of vascular disease or unexplained sudden death < 60 years [7]. All 51 patients included in this retrospective study were deemed suitable candidates for the FET procedure, thus excluding those managed with endovascular approaches [8].

Additional cardiovascular procedures were listed as considered necessary. The total number of patients is provided in Supplement S1. Data from HTAD patients treated with the FET technique were analyzed using our institutional database.

All surviving patients attended their 3-month follow-up appointment. Follow-up visits were scheduled in accordance with the 2024 EACTS/STS guidelines and adjusted as needed based on the underlying genetic variant and aortic disease.^1^ Comprehensive data collection, including external medical reports, computed tomography (CT) scans, and subsequent aortic interventions was documented during each visit. Follow-up data were analyzed retrospectively. The analysis adheres to the STrengthening the Reporting of OBservational studies in Epidemiology (STROBE) reporting criteria [9] and complies with the STROBE checklist.

### Definitions

Patients in this study were categorized according to the underlying gene harboring the pathogenic variants responsible for HTAD in line with the 2024 EACTS/STS guidelines [1]. Those diagnosed with acute aortic syndromes (AAS) underwent emergency surgery as indicated per EACTS guidelines. Elective operations include chronic aortic dissection and classical aneurysms of the aortic arch or proximal descending aorta. All surgeries of patients with previous sternotomies were categorized as reoperations. The FET implantation was defined as the primary surgery. Subsequent interventions refer to any thoracic aortic intervention performed after FET implantation. For elective operations, reoperations, and planned subsequent interventions following FET implantation, we adhered to the 2024 EACTS guidelines [1]. These guidelines use aortic growth rate and overall diameter over time as indicators for subsequent procedures, which were adapted for each patient based on post-operative or consequent follow-up CT findings. For symptomatic aneurysms, subsequent interventions were expedited as necessary.

Short-term survival included operative mortality, which was defined as any in-hospital deaths or deaths occurring within the first 30 days following the primary surgery. Emergency surgery was defined as a procedure performed within 24 h of diagnosis. Ventilation time was documented in hours, from arrival in the Intensive Care Unit (ICU) until extubation. Length of ICU stay included both the admission and discharge days. Acute kidney failure was noted if ongoing dialysis was required after discharge from the ICU. Neurological complications were documented if symptoms persisted until hospital discharge and were categorized according to the “modified Rankin Scale (mRS)”. Isolated neurological deficits of the lower extremity not attributable to stroke were defined as paraplegia. Recurrent nerve palsy diagnoses were made by the Ear Nose and Throat Department. Death occurring after hospital discharge was defined as late mortality. 30-Day and mid-term device-related complications encompass new tears induced by the distal end of the hybrid stent graft prosthesis (FET), specifically defined as distal stent graft-induced new entry (dSINE). This definition excludes tears arising from the underlying pathology. Mid-term survival includes outcomes measured 30 days postoperatively and thereafter.

### FET techniques

Before 2015, all patients treated at our Aortic Center underwent conventional FET procedures with distal anastomosis in aortic arch zone 3. In 2015, we adapted the conventional FET technique and implemented the innovative “simplified technique” involving the strategic proximalization of the distal anastomosis to arch zone 2. In 2019, Detter et al. published an extensive study detailing this “simplified technique” for FET implantation [10]. Alongside the FET implantation, aortic root replacement was performed using either the technique described and adjusted by David or Bentall. Following surgery, all patients were transferred to the ICU.

### Stent graft sizing and length

In chronic aortic dissection, distal endograft sizing was guided by Thoracic Endovascular Aortic Repair (TEVAR)-specific strategies, selecting grafts by crescent length without oversizing to minimize distal radial force and dSINE risk. In degenerative aneurysms, standard 10–20% oversizing relative to the perpendicular diameter was applied when distal sealing was feasible, though sizing was less critical if further distal repair was anticipated [11–13].

Stent-graft length was tailored to patient morphology and treatment strategy. In some pathologies 100 mm length may be sufficient for distally sealing certain pathologies in the mid-arch, while for further distal arch aneurysms a 150 mm length may be required to provide sufficient seal. In cases requiring distal extension with TEVAR length was determined depending on the needed overlap with the planned distal TEVAR components.

### Anticoagulation protocol

We restrict intraoperative coagulation product use to ROTEM- (rotational thromboelastometry)- guided indications. In patients without postoperative bleeding, therapeutic anticoagulation is initiated with low-molecular-weight heparin (dose-adjusted to factor Xa activity) and transitioned to a DOAC (direct oral anticoagulant) before discharge.

### Follow-up imaging

Over the 14 years, our diagnostic strategy evolved from minimizing radiation in younger patients to emphasizing early in-hospital CT for detecting device-related outcomes such as dSINE and intraluminal thrombosis, with imaging performed before discharge, sometimes as early as 1–2 days postoperatively. CT follow-up is performed at 3, 6, and 12 months postoperatively, with subsequent imaging guided by prior findings.

### Statistical analysis

Statistical analysis was conducted using IBM SPSS Version 27.0.1.0. Due to the small sample sizes, we presented the data descriptively. Baseline characteristics were presented as frequencies (n) and corresponding percentages (%), while perfusion times were expressed as mean ± SD. Due to the small patient numbers, age, operative, and outcome data, were reported in median and range. Survival and time-related rates of subsequent interventions were estimated using the Kaplan-Meier method.

### Ethical statement

The authors are accountable for all aspects of the research, ensuring that inquiries regarding the accuracy or integrity of any part of the study are appropriately addressed and resolved. The study was conducted following the Declaration of Helsinki (2013 revision). Data acquisition was performed anonymized and retrospectively. Under German legislation, ethical approval was not required, and patient consent was waived under § 12 HmbKHG (Hospital Law of Hamburg) and § 15 of the Medical Association’s professional code of conduct.

## Results

### Baseline characteristics

Of the 280 patients who underwent aortic arch repair using the FET technique, 51 patients diagnosed with HTAD were included in the study (Table 1). Median age was 43 years. MFS was the most prevalent (*n* = 31), followed by LDS (*n* = 11), nsHTAD (*n* = 8), and vEDS (*n* = 1). Most patients had normal preoperative renal (*n* = 43; 84.3%) and left ventricular function (*n* = 39; 76.5%). Comorbidities included arterial hypertension in 40 patients (78.4%), COPD requiring medical treatment in 5 (9.8%), and active smoking in 9 patients (17.6%).

**Table 1:** Baseline characteristics of HTAD cohorts

Characteristic	All patients *n* = 51	MFS*^1^ *n* = 31 (60.8%)	LDS*^2^ *n* = 11 (21.6%)	vEDS*^3^ *n* = 1 (2.0%)	nsHTAD*^4^ *n* = 8 (15.7%)
**Baseline**					
Age [y]*^5^	43 [14–60]	43 [14–60]	37 [20–50]	53	48 [27–60]
Male sex n (%)	25 (52.1)	17 (60.7)	3 (27.3)	1 (100)	4 (50)
Genetics (n)		FBN1 = 31	TGFBR1 = 2; TGFBR2 = 6; SMAD3 = 2; TGFB2 = 1	COL3A1	ACTA2 = 5; MYH11 = 2; MLYK = 1
Arterial Hypertension n (%)	40 (78.4)	26 (83.9)	7 (63.6)	0	7 (87.5)
Hyperlipidemia n (%)	8 (15.7)	5 (16.1)	0	0	3 (37.5)
Diabetes mellitus n (%)	2 (3.9)	1 (3.2)	1 (9.1)	0	0
Smoking (active) n (%)	9 (17.6)	3 (9.7)	1 (9.1)	0	5 (62.5)
COPD n (%)	5 (9.8)	3 (6)	1 (2)	0	1 (12.5)
Left ventricular function > 55%	39 (76.5)	24 (77.4)	9 (81.8)	1	5 (62.5)
Renal function normal n (%)	43 (84.3)	26 (83.9)	10 (90.9)	1	6 (75)
Reoperation (sternotomy) n (%) aortic root n (%) ascending aorta n (%) aortic arch n (%)	26 (51.0) 22 (43.2) 3 (5.9) 1 (2.0)	19 (61.3) 17 2 0	4 (36.4) 3 2 0	0	3 (37.5) 2 0 1
**Pathology**					
AAS (aTAAD, aTBAD)*^6^ n (%)	12 (23.5)	7 (22.6)	3 (27.3)	0	2 (25)
Chronic aortic dissection n (%)	32 (62.7)	20 (64.5)	7 (63.6)	1	4 (50)
Thoracic aortic aneurysm n (%)	7 (13.7)	4 (12.9)	1 (9.1)	0	2 (25)
**Urgency**					
Elective/urgent surgery n (%)	43 (84.3)	26 (83.9)	9 (81.8)	1	7 (87.5)
Emergency surgery^*7^ n (%)	8 (15.7)	5 (16.1)	2 (18.2)	0	1 (12.5)

*^1^Marfan Syndrome, *^2^Loeys-Dietz Syndrome, *^3^vascular Ehlers-Danlos Syndrome, *^4^Non-Syndromic HTAD (Hereditary Thoracic Aortic Disease), *^5^median [range], *^6^AAS (acute aortic syndrome including acute Type A (aTAAD) and B (aTBAD) Aortic, ^*7^operated within 24 h Dissection)

A total of 32 patients (62.7%) underwent elective surgery for chronic aortic dissection, while 7 patients (13.7%) had surgery true for thoracic aortic aneurysms, and the remaining 12 patients (23.5%) underwent surgery for AAS, 8 (15.7%) of which were operated as an emergency within 24 h after diagnosis. Among these HTAD patients, over 50% were reoperations (*n* = 26; 51.0%). These patients initially underwent surgeries that included aortic root (*n* = 22), ascending aorta (*n* = 3), and aortic arch (*n* = 1). The sole vEDS patient in our cohort underwent FET as primary surgery. A comparison of baseline characteristics between HTAD cohorts can be seen in Table 1.

### Operative data

The treatment of the proximal aorta involved concomitant aortic root replacement (*n* = 13; 25.5%) or supracoronary aortic replacement (*n* = 38; 74.5%). Before 2015, the FET was implanted in zone 3 (*n* = 13; 25.5%), thereafter, exclusively in zone 2 (*n* = 38; 74.5%). The vast majority (*n* = 50, 93.2%) of all implanted hybrid stent graft prostheses was the Terumo Aortic Thoraflex™ Hybrid Plexus prosthesis (Terumo Aortic, Inchinnan, Scotland, UK). Only the first patient of the cohort received the Jotec E-vita open plus hybrid prosthesis (Jotec, Hechingen, Germany). The stent graft length was either 100 mm (*n* = 28; 56.0%) or 150 mm (*n* = 18; 36.0%). For 5 (8.9%) patients the stent graft length was not documented. Depending on the pathology, stent graft diameter varied from 24 to 40 mm. Operative data and stent graft sizing for pathological subtypes are summarized in Tables 2 and 3, respectively.

**Table 2:** Operative data of HTAD cohorts

Variable	All *n* = 51	MFS*^1^ *n* = 31 (60.8%)	LDS*^2^ *n* = 11 (21.6%)	vEDS*^3^ *n* = 1 (2.0%)	nsHTAD*^4^ *n* = 8 (15.7%)
**Surgical details n (%)**					
Proximal					
supracoronary	38 (74.5)	24 (77.4)	5 (45.5)	1 (100)	8 (100)
valve-sparing root replacement	10 (19.6)	6 (19.4)	4 (36.4)	0	0
Bentall procedure	3 (5.9)	1 (3.2)	2 (18.2)	0	0
Aortic arch n (%)					
zone 3 (before 2015)	13 (25.5)	11 (35.5)	2 (18.2)	0	0
zone 2 (since 2015)	38 (74.5)	20 (64.5)	9 (81.8)	1 (100)	8 (100)
**Implanted devices n (%)**					
Thoraflex™ Hybrid Prosthesis	50 (98,0)	30 (96.7)	11 (100)	1 (100)	8 (100)
Jotec E-vita open Prosthesis	1 (1.9)	1 (3.2)	0	0	0
Stent graft 100 mm*^5^	28 (56)	18 (36)	4 (8)	1 (2)	5 (10)
Stent graft 150 mm*^5^	18 (36)	10 (20)	5 (10)	0	3 (6)
Stent graft diameter*^5,6^	28 [24–40]	28 [24–40]	26 [24–34]	24	28 [24–36]
Perfusion times [min]* ^**7**^					
Cardio-pulmonary bypass	253 ± 73	244 ± 71	288 ± 56	342	220 ± 55
Cardiac ischemia	125 ± 55	127 ± 55	127 ± 65	151	106 ± 31
Cerebral perfusion	71 ± 20	71 ± 21	78 ± 22	58	65 ± 13
Hypothermic circulatory arrest	48 ± 17	50 ± 19	50 ± 13	37	38 ± 8

*^1^Marfan Syndrome, *^2^Loeys-Dietz Syndrome, *^3^vascular Ehlers-Danlos Syndrome, *^4^Non-Syndromic HTAD (Hereditary Thoracic Aortic Disease), *^5^data incomplete, *^6^median [range], *^7^mean±standard deviation

**Table 3:** Stent graft sizing: pathological subtypes

	AAS^*1^ *n* = 12 (23.5%)	CAD^*2^ *n* = 32 (62.7%)	Aneurysm *n* = 7 (13.7%)
**Stent graft length 100 mm** ^*3^ **Stent graft length 150 mm** ^*3^	10 2	13 15	5 1
Stent graft diameter*^3^ **[mm]** median [range]	28 [24–32]	28 [24–36]	28 [24–40]

*^1^AAS (Acute Aortic Syndrome including acute Type A (aTAAD) and B (aTBAD) Aortic Dissection, ^*2^CAD (Chronic Aortic Dissection), *^3^incomplete data

### 30-day outcomes

#### Survival

The overall 30-day survival rate was 98.0% with 50 out of 51 patients surviving. There was only one in-hospital death attributed to stroke following emergency surgery for AAS. The survival rate for emergency and elective patients (including those who underwent reoperation), was 87.5% (*n* = 7/8) and 100% (*n* = 43/43), respectively (Table 4).

**Table 4:** 30-Day and Mid-term outcomes of HTAD groups

Outcome	All *n* = 51	MFS*^1^ *n* = 31 (60.8%)	LDS*^2^ *n* = 11 (21.6%)		vEDS*^4^ *n* = 1 (2.0%)	nsHTAD*^3^ *n* = 8 (15.7%)
**30-Day Outcome**						
**Survival n (%)**						
Overall *(including Reoperation)*	50 (98.0) *26 (100)*	30 (96.8) *19 (100)*	11 (100) *4 (100)*		1 (100) *0*	8 (100) *3 (100)*
Elective	43 (100)	26 (100)	9 (100)		1 (100)	7 (100)
Emergency	7 (87.5)	5 (83.3)	2 (100)		0	1 (100)
** In-Hospital Data**						
Overall stay [days]*^6^	13 [3–108]	13 [3–108]	12 [7–87]		13	16 [7–48]
ICU stay [days]*^6^	4 [1–56]	4 [1–49]	4 [1–56]		4	6 [1–48]
Ventilation time [hours]*^6^	15 [3–837]	15 [3–620]	14 [4–29]		17	13 [6–837]
Renal failure n (%)	5 (10.0)	2 (6.5)	1 (2.0)		0	2 (4.0)
Re-thoracotomy n (%)	3 (5.9)	2 (6.5)	0		0	1 (12.5)
Recurrent nerve palsy n (%)	9 (17.6)	4 (12.9)	3 (27.5)		0	2 (25.0)
Neurological						
stroke (permanent) n (%)	2 (3.9)	1 (3.2)	1 (9.0)		0	0
modified Ranking scale		1	2			
Paraplegia n (%)	0	0	0		0	0
Midterm Follow-up n*^7^	*n* = 50	*n* = 30	*n* = 11		*n* = 1	*n* = 8
Period*^6^		4 years [1.1–148] months				
Survival n (%)	46 (92)	27 (90)	10 (91)		1 (100)	8 (100)
Cause of death						
cerebral bleeding n	1	0	1		0	0
heart failure n	1	1	0		0	0
unknown n	1	1	0		0	0
acute pancreatitis n	1	1	0		0	0
**Subsequent thoracic aortic interventions n (%)**	**24 (82.7)**					
TEVAR n (%)	19 (65.5)	12 (63.2)	5 (71.4)		0	2 (66.7)
Crawford operation n (%)	5 (17.2)	5 (26.3)	0		0	

*^1^Marfan Syndrome, *^2^Loeys-Dietz Syndrome, *^3^vascular Ehlers-Danlos Syndrome, *^4^Non-Syndromic HTAD (Hereditary Thoracic Aortic Disease), *^4^vascular Ehlers-Danlos Syndrome, *^5^data unavailable, *^6^median [range], *^7^ data incomplete

#### Procedure-related outcomes

The median ventilation time and length of ICU stay were 15 h and 4 days, respectively. Acute renal failure was observed in 5 patients (10%) and re-thoracotomy was performed in 3 patients (5.9%). One patient was diagnosed with permanent neurological deficit, severity level 2 on the modified Rankin scale (mRS). A second patient initially presented with neurological deficit, but was discharged without any impairments, scoring mRS 1. Paraplegia did not occur in any of the patients and recurrent nerve palsy was identified in 9 (17.6%).

#### Early device-related outcomes

A complete CT follow-up was only available in 32 of 51 patients (62.7%). Intraluminal FET thrombosis was observed in 4 patients (12.5%), while distal stent graft-induced new entry (dSINE) was identified in 3 patients (9.4%). Incomplete early postoperative CT follow-up in 32 of 51 patients was not significantly associated with 30-day (*p* = 0.34) or 6-month mortality (*p* = 0.72) in those with intraluminal FET thrombosis. A summary of 30-day device-related complications is provided in Table 5.

**Table 5:** 30-Day and Mid-term device related complications:

30-Day device related complications					
dSINE*^1^ **n = 3 (9.4%)**	**Genetic variant**	**Indication** **for surgery**	POD* ^**2**^	**Treatment**	**Outcome**
1 (3.1); asymptomatic	FBN1*^3^	AAS*^4^	14 24	emergency TEVAR subsequent Crawford	successfully treated
1 (3.1); symptomatic	TGFBR1	CAD*5	1	emergency TEVAR	successfully treated
1 (3.1); symptomatic	TGFBR2	CAD	4	emergency TEVAR	successfully treated
Intraluminal FET thrombosis **n = 4 (12.5%)**					
1 (3.1); asymptomatic	SMAD3	CDA	10	Conservative Anticoagulation, vitamin K antagonists	successfully treated
1 (3.1); asymptomatic	ACTA2	AAS	6	Conservative Anticoagulation, vitamin K antagonists	successfully treated
1 (3.1); kidney failure	FBN1	CAD	1	TEVAR	successfully treated
1 (3.1) asymptomatic	FBN1	AAS	5	Conservative Anticoagulation, vitamin K antagonists	successfully treated
Mid-term device related complications*^7^					
dSINE*^1^ **n = 2 (4.3%)**	**Genetic variant**	**Indication** **for surgery**	POM* ^**6**^	**Treatment**	**Outcome**
1 (2.1); asymptomatic	FBN1	AAS	12	TEVAR	successfully treated
1 (2.1); asymptomatic	TGFBR2	CAD	8	TEVAR	successfully treated

^*1^ distal Stentgraft Induced New Entry, ^*2^ postoperative day, ^*3^ neonatal Marfan syndrome, *^4^ acute aortic syndrome, *^5^ chronic aortic dissection, ^*6^ postoperative month, *^7^incomplete data (follow-up CTs available in 47 out of 50 patients (94%)

### Mid-term outcomes

#### Survival

**Fig. 1:**
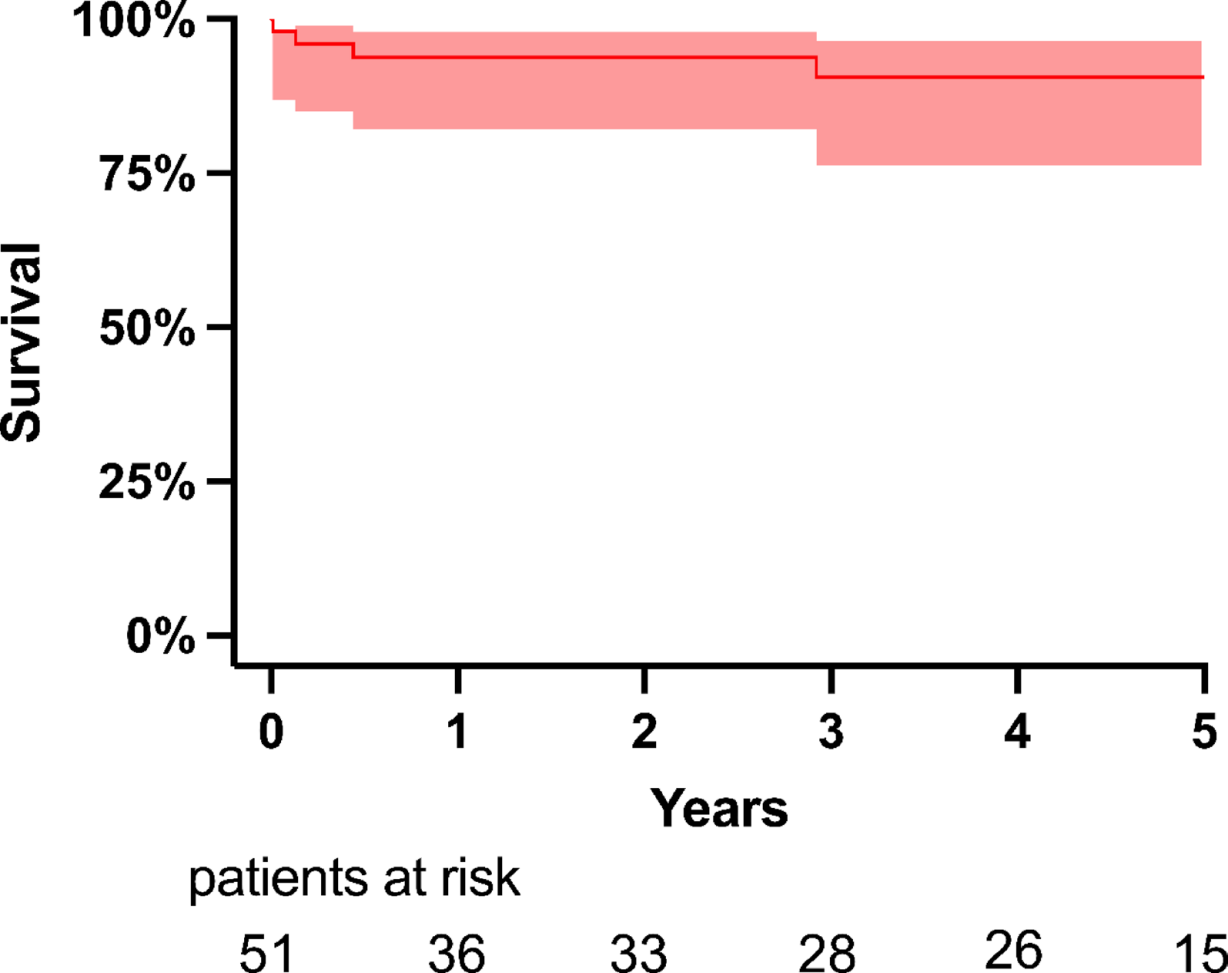
Survival

We achieved a 100% 3-month follow-up of surviving patients. During a median follow-up duration of 48 [1.1 to 148] months, the mid-term overall survival rates at 1-, 3- and 5-year were 93.9%, 90.6%, and 90.6%, respectively (Fig. 1). Most late deaths occurred in the MFS group, accounting for 11.1% (*n* = 3/31), and the LDS group, accounting for 10% (*n* = 1/11).

#### Mid-term device-related outcomes

CT imaging was incomplete and available for 47 of the 50 surviving patients. In addition to the early 3 (9.4%) dSINE patients, another 2 (4.3%) patients were diagnosed and treated for a dSINE, one MFS patient at 12 months, and one LDS patient at 8 months. Mid-term device-related complications are summarized in Table 5.

#### Subsequent interventions

**Fig. 2:**
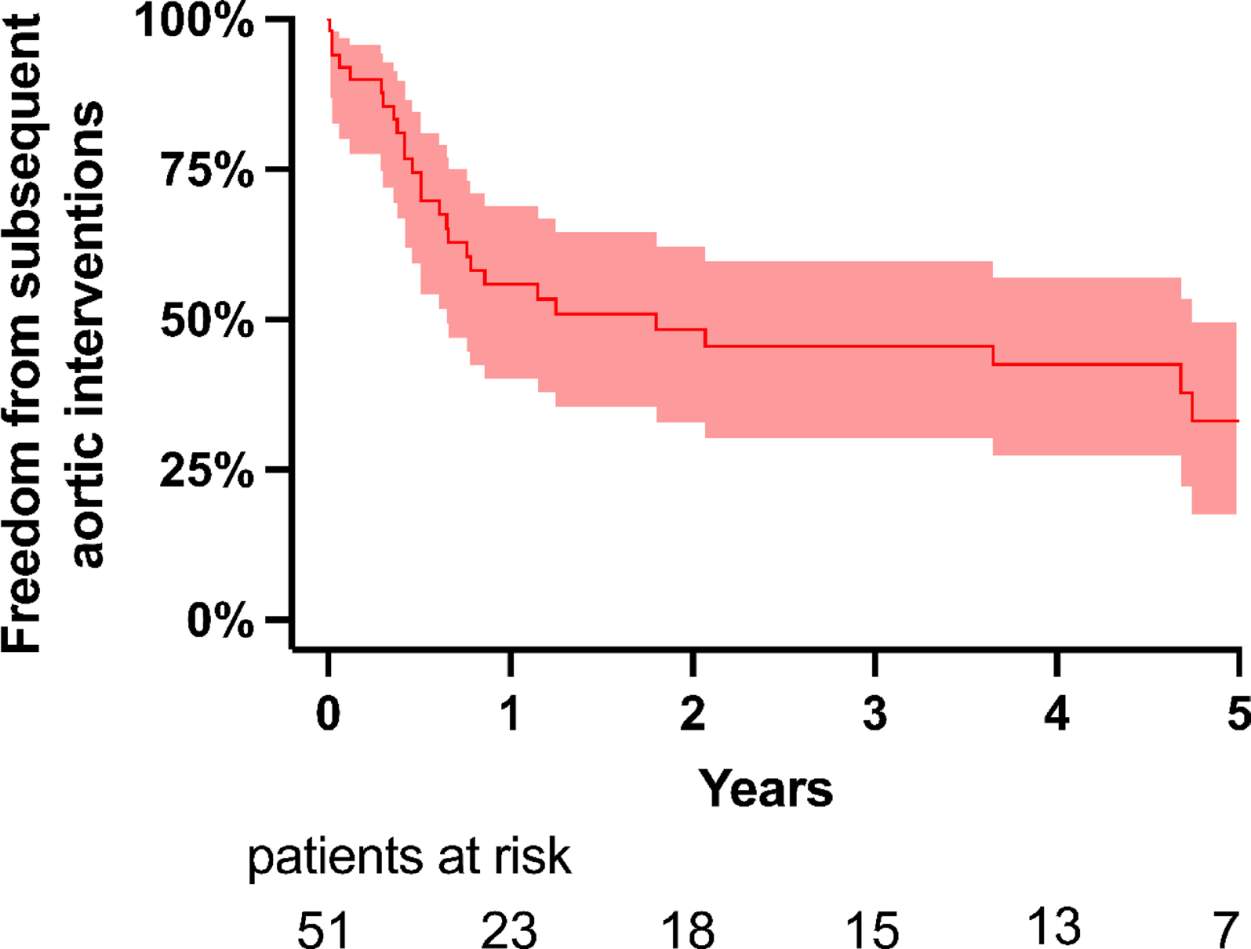
Freedom from reintervention

As anticipated, our HTAD cohort had a high rate of subsequent interventions at 1, 3, and 5 years 32.7%, 44.9%, and 47.0%, respectively (Fig. 2). The nsHATD group showed the nominal highest incidence at 66.7% (2/8), followed by MFS at 56.6% (17/30), LDS at 45.5% (5/11), and none for vEDS. None of the subsequent interventions were performed as emergency surgery.

The most frequent intervention after the primary FET implantation was TEVAR (*n* = 19; 38.0%), of which *n* = 2 (4.0%) was combined with the Candy-Plug technique (false lumen endograft). Open surgical replacement of the descending aorta as described by Crawford was performed in 4 patients (8.3%). The Thoracoflo™ prosthesis (Terumo Aortic, Inchinnan, Scotland, UK) was implanted in 2 (4.2%) of the 4 Crawford patients. Early, 30-day, and mid-term operative outcomes are summarized in Table 4.

## Discussion

### Main findings

This single-center study identified 51 patients with HTAD who underwent FET procedures with hybrid stent graft prostheses and includes a representation of patients across all categories of HTAD^1^. Elective patients (*n* = 43) yielded 100% 30-day survival and low overall complications. FET as reoperation in elective patients, yielded 100% 30-day survival. Overall mid-term survival at 5 years was 90.9%. The main findings of this study are summarized in Fig. 1.

### 30-day outcomes

Compared to the 100% survival among our elective surgery patients, the 30-day survival rate for the emergency treatment group was 87.5% (7 out of 8). Other studies demonstrate lower survival rates in the treatment of both AAS and chronic dissection [2, 3, 14]. We also report a 100% survival rate for aortic arch replacement as reoperations, contrary to the results in other studies [15, 16]. We observed short ventilation times and length of ICU stay, low incidence of neurological complications (3.9%) and renal failure (10%), and no occurrences of paraplegia.

Intraluminal thrombosis causing thromboembolic events and malperfusion after FET implantation is said to be associated with unfavorable outcomes [17]. Risk factors contributing to intraluminal thrombosis following FET surgery are reported to include female gender, extensive aneurysm, advanced age, under-sizing of the stent graft leading to type 1b endoleak which can precipitate low flow and stasis at the transition stent graft native aorta [18]. In our cohort, the incidence of device-related complications was low, with four patients (12.5%) presenting with early intraluminal thrombosis and three with dSINE (9.4%). The low occurrence of intraluminal thrombosis observed in our HTAD cohort may be attributed to the predominantly younger, male patients and a lower prevalence of aneurysms. Furthermore, the low early dSINE incidence might be due to the predominance of chronic aortic dissections with small true lumens, requiring smaller stent graft sizes. Our findings align with Czerny et al., who noted early dSINE due to oversizing in acute or chronic dissections [19].

### Mid-term outcomes

The overall mid-term mortality was 8.0% (*n* = 4). One MFS patient succumbed to heart failure, another died of pancreatitis, one MFS patient’s death was of unknown cause, and an LDS patient died of a cerebral hemorrhage. In contrast, no deaths were observed for either nsHTAD patients or the one vEDS patient. Survival rates at 3 and 5 years postoperatively remained consistently stable in our cohort, at 90.9%.

Long-term survival from external centers shows significant variation. In HTAD patients it is reported to be directly related to the progression of aortic or cardiovascular disease [20, 21], for example, heart failure accompanied by cardiac arrhythmias in MFS [22] and the presence of intracranial aneurysms as shown among LDS patients [23]. Follow-up studies particularly focusing on HTAD cohorts and patients under 30 years, report 5-year survival rates after AAS and chronic dissection ranging from 70% to 85.5% [2, 14, 16, 24], with chronic dissection yielding better outcomes.^3^ Survival rates in our cohorts of HTAD with AAS, chronic dissection, and aneurysm exceeded 90%.

In patients with HTAD, subsequent surgery is generally associated with reduced long-term survival. In our cohort, mid-term survival after subsequent interventions was not adversely affected, as no deaths occurred despite the relatively high frequency of reinterventions. In our cohort, mid-term survival after subsequent interventions was not adversely affected, with no deaths observed despite their relatively high frequency [3, 25]. Open surgical treatment according to Crawford and endovascular therapy both offer reliable outcomes. Crawford’s approach benefits from the anastomosis site being moved distally, while the latter benefits from an optimal proximal landing zone to prevent secondary endoleaks [26, 27]. Compared to the reported rates of Czerny et al. (6.5%) [19] and Murana et al. (12.7%) [28], we observed a low incidence of mid-term dSINE (4.3%), like our early follow-up.

The promising overall short- and mid-term results from our single-center study are likely due to the following factors: (1) well-established aortic surgical team, consisting mainly of two surgeons for most of our “simplified FET technique”, ensuring continual precision and refinement [10], (2) stabilizing the proximal descending aorta with the FET technique using commercially available prostheses, (3) providing an ideal landing zone for subsequent interventions i.e., TEVAR, with or without insertion of a Candy-Plug,^29^ and Crawford surgery with or without ThoracoFlo™ hybrid graft implantation [30] and, (4) regular follow-up and collaborative operative decision-making within the Multidisciplinary Aortic Team, following the 2023 EACTS/STS guidelines [1, 31].

### Limitations

This study is limited by its retrospective, single-center design, small sample size, and heterogeneity of pathologies. Another limitation is the incomplete follow-up CT data during the 14-year period, which restricted the assessment of early device-related outcomes such as dSINE and intraluminal thrombosis. This was partly due to an initial strategy to minimize lifetime radiation exposure in younger patients, before subsequent protocol modifications introduced routine early postoperative CT imaging prior to discharge. The use of a single device type in nearly all our patients (50 out of 51) restricts the clinical applicability and generalizability of the findings. This study evaluates short- and mid-term outcomes of hybrid stent grafts in FET procedures for patients with HTAD, focusing on safety and feasibility. Hence, conclusions on long-term durability and effectiveness cannot be drawn. MFS and LDS represent two extensively researched HTAD syndromes, while the nsHTAD group is underrepresented.

## Conclusion

Our analysis indicates that commercially available hybrid stent graft prostheses can be safely implanted using the FET technique in the treatment of both elective and acute HTAD patient cohorts with pathologies of the aortic arch and proximal descending aorta. Short- and mid-term outcomes are encouraging, with no fatal aortic events and low rates of device-related complications. The findings support the safety and feasibility of FET procedures in patients with HTAD; however, they do not confirm the long-term durability and efficacy of hybrid stent graft prostheses. Further validation and long-term follow-up in larger-scale studies and registries are warranted.

## Supplementary Information

Supplementary Material 1

## Data Availability

No datasets were generated or analysed during the current study.

## References

[1] Czerny M, Berger T, Della Corte A et al. Clinical cases referring to the 2023 EACTS/STS guidelines for diagnosing and treating acute and chronic syndromes of the aortic organ. Eur J Cardiothorac Surg. 2024;66(3): 1-100 10.1093/ejcts/ezae294 39196761

[2] Widenka KJ, Kosiorowska M, Jakob H, et al. Early and midterm results of frozen elephant trunk operation with Evita open stent-graft in patients with Marfan syndrome: results of a multicentre study. BMC Cardiovasc Disord. 2022;22(1):333. 35883019 10.1186/s12872-022-02777-5 PMC9317434

[3] Chen Y, Ma WG, Li JR, et al. Is the frozen elephant trunk technique justified for chronic type A aortic dissection in Marfan syndrome? Ann Cardiothorac Surg. 2020;9(3):197–208. 32551252 10.21037/acs.2020.03.10 PMC7298250

[4] Uchida N, Katayama A, Kuraoka M, et al. Extended aortic repair using frozen elephant trunk technique for Marfan syndrome with acute aortic dissection. Ann Thorac Cardiovasc Surg. 2013;19(4):279–82. 23196664 10.5761/atcs.oa.12.01930

[5] Roselli EE, Idrees JJ, Lowry AM, et al. Beyond the aortic root: staged open and endovascular repair of arch and descending aorta in patients with connective tissue disorders. Ann Thorac Surg. 2016;101(3):906–12. 26545624 10.1016/j.athoracsur.2015.08.011

[6] Preventza O, Mohammed S, Cheong BY. Endovascular therapy in patients with genetically triggered thoracic aortic disease: applications and short- and mid-term outcomes. Eur J Cardiothorac Surg. 2014;46(2):248–53. 24477738 10.1093/ejcts/ezt636

[7] von Kodolitsch Y, Rybczynski M, Vogler M, et al. The role of the multidisciplinary health care team in the management of patients with Marfan syndrome. J Multidiscip Healthc. 2016;9:587–614. 27843325 10.2147/JMDH.S93680 PMC5098778

[8] Eleshra A, Panuccio G, Spanos K, et al. Safety and effectiveness of TEVAR in native proximal landing zone 2 for chronic type B aortic dissection in patients with genetic aortic syndrome. J Endovasc Ther. 2022;29(5):717–23. 34894819 10.1177/15266028211061276

[9] von Elm E, Altman DG, Egger M, et al. The strengthening the reporting of observational studies in epidemiology (STROBE) statement: guidelines for reporting observational studies. J Clin Epidemiol. 2008;61(4):344–9. 18313558 10.1016/j.jclinepi.2007.11.008

[10] Detter C, Demal TJ, Bax L, et al. Simplified frozen elephant trunk technique for combined open and endovascular treatment of extensive aortic diseases. Eur J Cardiothorac Surg. 2019;56(4):738–45. Erratum in: Eur J Cardiothorac Surg. 2019;56(4):817. 30957865 10.1093/ejcts/ezz082

[11] Dong Z, Fu W, Wang Y, Wang C, et al. Stent graft-induced new entry after endovascular repair for Stanford type B aortic dissection. J Vasc Surg. 2010;52(6):1450–7. 20800417 10.1016/j.jvs.2010.05.121

[12] Rohlffs F, Grandi A, Panuccio G, et al. Endovascular options for the ascending aorta and aortic arch: a scoping review. Ann Vasc Surg. 2023;94:102–18. 37328096 10.1016/j.avsg.2023.06.004

[13] Ogawa Y, Nishimaki H, Chiba K, et al. Clinical utility of the Candy-Plug technique using an excluder aortic extender. Ann Vasc Dis. 2021;14(2):139–45. 34239639 10.3400/avd.oa.21-00018 PMC8241552

[14] Xie Q, Zhong Y, Xu Q, et al. Early and long-term outcomes of young adult patients ≤ 30 years old with acute type A aortic dissection. Eur J Cardiothorac Surg. 2023;64(6):ezad330. 37758246 10.1093/ejcts/ezad330

[15] Bachet J, Larrazet F, Goudot B, et al. When should the aortic arch be replaced in Marfan patients? Ann Thorac Surg. 2007;83(2):S774–9. ;discussion S785-90. 17257925 10.1016/j.athoracsur.2006.10.085

[16] Chen Y, Ma WG, Zheng J, et al. Total arch replacement and frozen elephant trunk for type A aortic dissection after Bentall procedure in Marfan syndrome. J Thorac Dis. 2018;10(4):2377–87. 29850143 10.21037/jtd.2018.03.79 PMC5949446

[17] Pastuszak Ż, Stępień A, Kordowska J, et al. Brain strokes related to aortic aneurysma - the analysis of three cases. Open Med (Wars). 2017;12:58–61. 28730163 10.1515/med-2017-0011 PMC5444340

[18] Helms F, Schmack B, Weymann A, et al. Risk factors, prevention, and therapy of intraluminal stent thrombosis in frozen elephant trunk prostheses-what we know so far. Front Cardiovasc Med. 2024;11:1344292. 38545343 10.3389/fcvm.2024.1344292 PMC10965621

[19] Czerny M, Eggebrecht H, Rousseau H, et al. Distal stent graft-induced new entry after TEVAR or FET: insights into a new disease from EuREC. Ann Thorac Surg. 2020;110(5):1494–500. 32283085 10.1016/j.athoracsur.2020.02.079

[20] Vanem TT, Rand-Hendriksen S, Brunborg C, et al. Health-related quality of life in Marfan syndrome: a 10-year follow-up. Health Qual Life Outcomes. 2020;18(1):376. 33256748 10.1186/s12955-020-01633-4 PMC7706277

[21] Mühlstädt K, De Backer J, von Kodolitsch Y, et al. Case-matched comparison of cardiovascular outcome in Loeys-Dietz syndrome versus Marfan syndrome. J Clin Med. 2019;8(12):2079. 31795342 10.3390/jcm8122079 PMC6947024

[22] Demolder A, Bianco L, Caruana M, et al. Arrhythmia and impaired myocardial function in heritable thoracic aortic disease: an international retrospective cohort study. Eur J Med Genet. 2022;65(6):104503. 35427808 10.1016/j.ejmg.2022.104503

[23] Huguenard AL, Johnson GW, Osbun JW, et al. Natural history and growth rate of intracranial aneurysms in Loeys-Dietz syndrome: implications for treatment. J Neurosurg. 2023;10:1–8. 10.3171/2023.8.JNS23733 37948688

[24] Luehr M, Yildiz M, Ma WG, et al. Acute type A aortic dissection in adolescents and young adults under 30 years of age: demographics, aetiology and postoperative outcomes of 139 cases. Eur J Cardiothorac Surg. 2023;63(5):ezad112. 36951534 10.1093/ejcts/ezad112

[25] Aranson NJ, Patel PB, Mohebali J. Presentation, surgical intervention, and long-term survival in patients with Marfan syndrome. J Vasc Surg. 2020;72(2):480–9. 32085956 10.1016/j.jvs.2019.10.060

[26] Kreibich M, Berger T, Walter T, et al. Downstream thoracic endovascular aortic repair following the frozen elephant trunk procedure. Cardiovasc Diagn Ther. 2022;12(3):272–7. 35800359 10.21037/cdt-22-99 PMC9253175

[27] Olsson KW, Mani K, Burdess A, et al. Outcomes after endovascular aortic intervention in patients with connective tissue disease. JAMA Surg. 2023;158(8):832–9. 37314760 10.1001/jamasurg.2023.2128 PMC10267845

[28] Murana G, Costantino A, Campanini F, et al. Distal stent graft-induced new entry (dSINE) after frozen elephant trunk: a scoping review. Cardiovasc Diagn Ther. 2023;13(2):408–17. 37583692 10.21037/cdt-22-234 PMC10423728

[29] Rohlffs F, Tsilimparis N, Fiorucci B, et al. The candy-plug technique: technical aspects and early results of a new endovascular method for false lumen occlusion in chronic aortic dissection. J Endovasc Ther. 2017;24(4):549–55. 28490232 10.1177/1526602817709252

[30] Debus ES, Malik K, Kölbel T, et al. First in human implantation of the Thoracoflo graft: a new hybrid device for thoraco-abdominal aortic repair. EJVES Vasc Forum. 2023;58:28–31. 37006724 10.1016/j.ejvsvf.2023.02.002 PMC10053397

[31] Andersen ND, Ganapathi AM, Hanna JM, et al. Outcomes of acute type a dissection repair before and after implementation of a multidisciplinary thoracic aortic surgery program. J Am Coll Cardiol. 2014;63(17):1796–803. 24412454 10.1016/j.jacc.2013.10.085 PMC4159705

