# Supplementary figures and images for: Hybrid prosthesis in frozen elephant trunk procedures for hereditary thoracic aortic diseases: a 14-year single-aortic center experience
Source: J Cardiothorac Surg. 2025 Nov 27;20:458. doi: 10.1186/s13019-025-03738-7 (PMC12699886; doi:10.1186/s13019-025-03738-7)

Identification

Screening

Inclusion

Follow-up

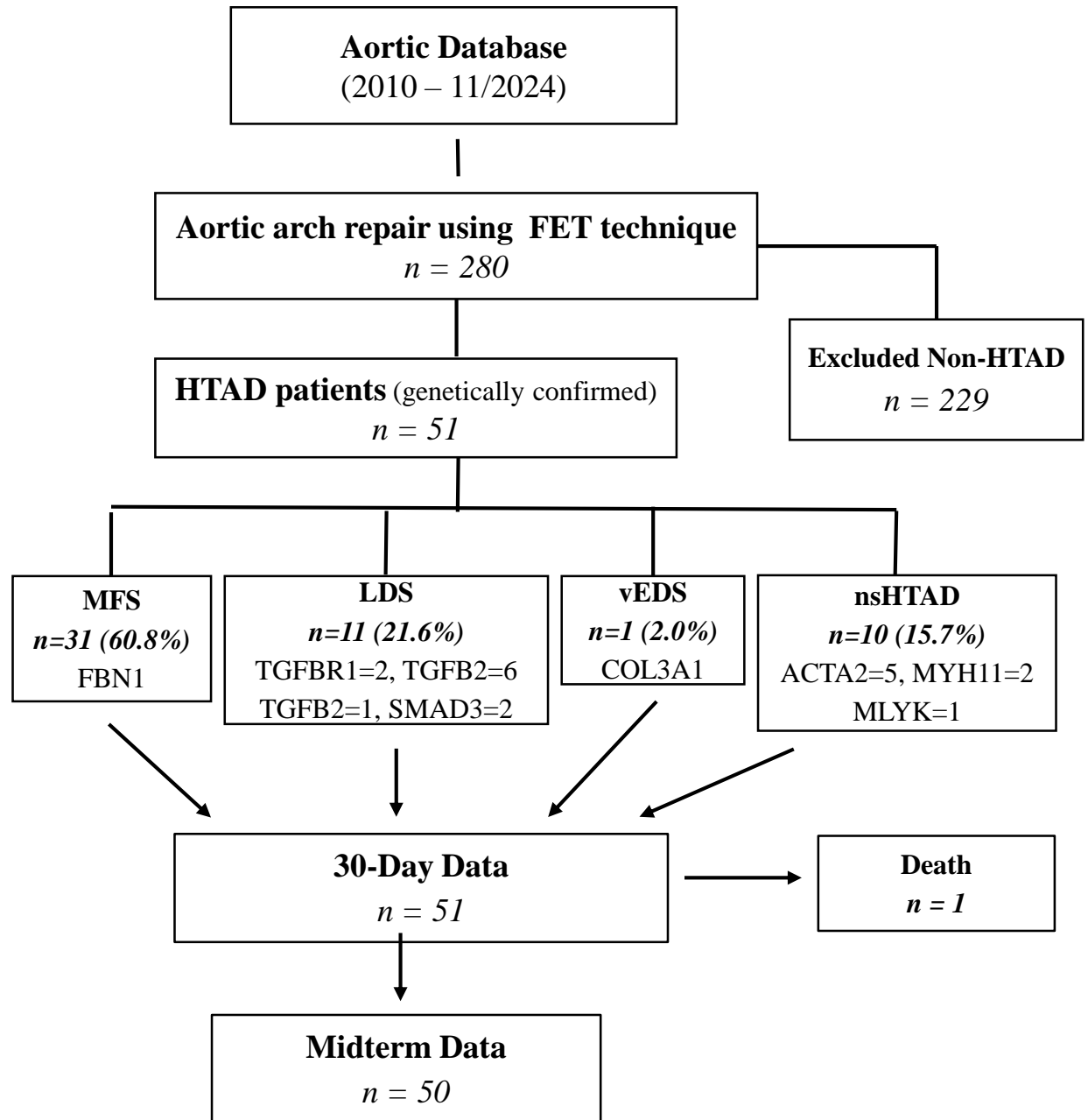

Supplement: Supplementary file 1 — Supplementary Material 1 [file 13019_2025_3738_MOESM1_ESM.pdf]
